# Exosomes from cervical cancer cells facilitate pro-angiogenic endothelial reconditioning through transfer of Hedgehog–GLI signaling components

**DOI:** 10.1186/s12935-021-02026-3

**Published:** 2021-06-24

**Authors:** Anjali Bhat, Joni Yadav, Kulbhushan Thakur, Nikita Aggarwal, Tanya Tripathi, Arun Chhokar, Tejveer Singh, Mohit Jadli, Alok Chandra Bharti

**Affiliations:** Molecular Oncology Laboratory, Department of Zoology, University of Delhi (North Campus), Delhi, 110007 India

**Keywords:** Cervical cancer, Exosome, Angiogenesis, Hh-GLI signaling, Tumor microenvironment, VEGF

## Abstract

**Background:**

Angiogenic switch is a hallmark feature of transition from low-grade to high-grade cervical intraepithelial neoplasia (CIN) in cervical cancer progression. Therefore, early events leading to locally-advanced cervical metastatic lesions demand a greater understanding of the underlying mechanisms. Recent leads indicate the role of tumor-derived exosomes in altering the functions of endothelial cells in cervical cancer, which needs further investigation.

**Methods:**

Exosomes isolated from cervical cancer cell lines were assessed for their angiogenic effect on the human umbilical vein endothelial cells (HUVEC) using tube formation and wound healing assay. The exosomal uptake by HUVEC cells was monitored using PKH-67 labelling followed by fluorescence microscopy. Alterations in Hh-GLI signaling components, PTCH1 and GLI1, in HUVEC were measured by immunoblotting. Changes in angiogenesis-related transcripts of vascular endothelial growth factor VEGF-A, VEGF-B, VEGFR2 and angiopoietin-1, angiopoietin-2, osteopontin were measured in exosome-treated HUVEC and in the exosomal RNA by RT-PCR.

**Results:**

Enhanced tube formation, with an increased number of nodes and branching was observed in HUVEC’s treated with exosomes derived from different cervical cancer cell lines. HPV-positive (SiHa and HeLa) cells’ exosomes were more angiogenic. Exosome-treated HUVEC showed increased migration rate. PKH-67 labelled exosomes were found internalized in HUVEC. A high level of PTCH1 protein was detected in the exosome—treated endothelial cells. Subsequent RT-PCR analysis showed increased transcripts of Hh-GLI downstream target genes VEGF-A, VEGFR2, angiopoietin-2, and decreased expression of VEGF-B, and angiopoietin-1, suggestive of active Hh-GLI signaling. These effects were more pronounced in HUVEC’s treated with exosomes of HPV-positive cells. However, these effects were independent of tumor-derived VEGF-A as exosomal cargo lacked VEGF-A transcripts or proteins.

**Conclusion:**

Overall, the data showed cervical cancer exosomes promote pro-angiogenic response in endothelial cells via upregulation of Hh-GLI signaling and modulate downstream angiogenesis-related target genes. The study provides a novel exosome-mediated mechanism potentially favoring cervical angiogenesis and thus identifies the exosomes as potential pharmacological targets against locally-advanced metastatic cervical lesions.

**Graphic abstract:**

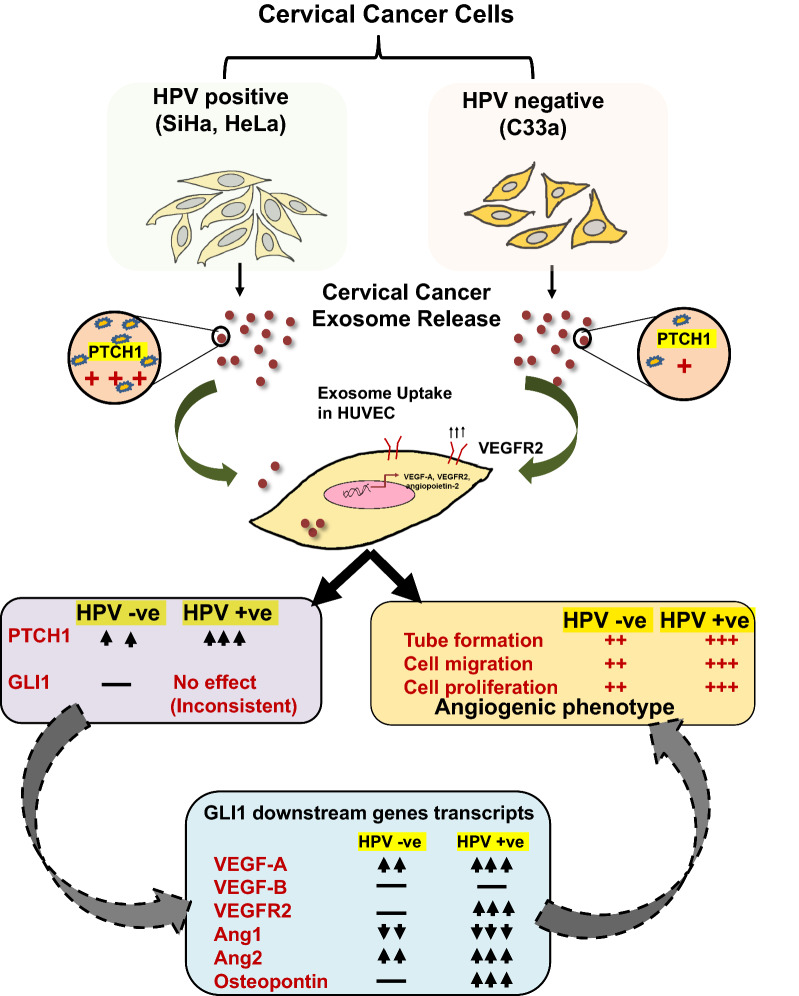

**Supplementary Information:**

The online version contains supplementary material available at 10.1186/s12935-021-02026-3.

## Background

Cervical cancer is caused by persistent infection of high risk human papillomavirus (HPV) and poses a major reproductive health challenge for women. Less developed countries disproportionately give rise to over 85% of the global disease burden [1]. High risk HPV types, HPV16 and HPV18 collectively contribute to over 80% of the invasive cervical cancers [2]. A detailed understanding of HPV biology over the last 50 years has improved our knowledge of cervical carcinogenesis, however, the disease is far from eliminated and ranked as the fourth top-most reported malignancy globally [3]. Cervical cancer is a treatable cancer but only when it is detected in the early stages, involving the identification of precursor and locally-advanced metastatic lesions. Invasive squamous cell carcinoma develops over a long period via initiation of precursor metastatic lesions [4], wherein productive interactions of cancer cells with their immediate environment determine the outcome. However, knowledge of these interactions is very limited and requires deeper understanding for developing therapeutics for management and control of tumor progression.

The tumor microenvironment is a major contributor to cervical cancer progression and witnesses a dynamic relationship between cervical cancer cells and their neighbors particularly, endothelial cells [5]. In this regard, activation of pro-angiogenic genes and neo-vascularization is an important event that marks the cervical intraepithelial neoplasia (CIN)-2 transition [6, 7] and subsequent cancer progression to advanced metastatic lesions. Therefore, angiogenesis has emerged as a therapeutic target for treatment of recurrent and metastatic cervical cancer [8]. Higher angiogenesis observed in cervical neoplasia was directly linked to VEGF expression by tumor cells [9] and it has emerged as a prognostic biomarker [10]. Targeting angiogenesis using anti-VEGF immunotherapy increased survival in advanced cervical cancer [11]. Cervical cancer cell lines were shown to release angiogenic modulators VEGF, bFGF, IL-8, TGF-β, and TNF-α in the conditioned medium [12]. HPV oncoproteins directly induced VEGF transcription [13] and resulted in angiogenic switch in primary keratinocytes to alter endothelial cell behavior [14]. How these mediators travel from source/tumor cells to their target endothelial cells is an area of active investigation. Moreover, VEGF immunotherapy is associated with the development of either evasive or adaptive resistance [15]. These observations clearly suggest a contribution of compensatory and alternate angiogenic mechanisms, which require deeper understanding.

Exosomes (30–100 nm) play context-dependent contrasting roles in cell-to-cell communication during angiogenesis [16]. Tumor-derived exosomes are mostly pro-angiogenic in nature [17] and in cervical cancer their secretion is upregulated [18]. HPV oncogenes, both E6 and E7, influence the content and extent of exosomal cargo in cervical cancer cells [19]. Among different RNA reported in the exosomal cargo of cervical cancer, microRNA-221 [20, 21] and lncRNA-TUG1 [22] have been implicated in tumor-associated angiogenesis. Interestingly, some of the important cell signaling components including receptors like EGFR [23], PTCH1, Shh, Ihh [24], Notch [25]; various ligands like, TNF-α [26], Wnt [27], Delta [28], Jagged-1 [29] and VEGF [30]; and signal transducers like p-Stat3 [31] have been reported in exosomal cargo. Among these, the presence of Hedgehog (Hh)-GLI signaling components was the most notable. Many of the genes associated with angiogenic response (VEGF, VEGFR, angiopoietin, osteopontin) are the direct downstream targets of Hh-GLI signaling [32].

Aberrant activation of the Hh-GLI pathway is an important oncogenic signaling pathway in many epithelial tumors [33]. Hh-GLI signaling plays a crucial role in pathogenesis and therapy responsiveness of cervical cancer [34, 35]. It has been suggested that HPV assisted Hh-GLI co-activation synergies to generate aggressive phenotype in cervical cancer cells [36]. Multiple classes of small molecules acting as Hh inhibitors are also shown to decrease growth of cervical cells [36, 37]. It is evident that Hh-GLI signaling is a critical mediator of cervical cancer tumor pathophysiology; however, the underlying mechanisms are poorly understood.

Earlier, we showed that cervical cancer cells possess an active Hh-GLI signaling, and HPV E6 played an instrumental role in its constitutive activation [36]. Subsequently, we found cervical cancer exosomes carrying large quantities of Hh-GLI signaling components like PTCH1 [24]. However, the functional importance of this observation was unknown. Therefore, the present investigation was designed to elucidate the ability of the cervical cancer exosomes in modulating the angiogenic phenotype and re-conditioning of the endothelial cells with special emphasis on Hh-GLI signaling and checked for differences with respect to the HPV status of cervical cancer cells.

## Materials and methods

### Materials

Human cervical cancer cell lines with known HPV positivity for HPV type 16—SiHa and HPV type 18—HeLa; and HPV-negative C33a were originally procured from ATCC, human umbilical vein endothelial cells (HUVEC; #CL002) were procured from HiMedia Laboratories Pvt Ltd. (Mumbai, India). The materials used in the study have been listed along with their source of procurement. DMEM (#AL111-18X500ML), MEM (#AT154), antibiotic–antimycotic solution (#A018), heat-inactivated fetal bovine serum (#RM10409), bovine serum albumin, fraction V (#RM10409) were procured from HiMedia. HiEndoXL™ endothelial cell expansion medium reduced serum (#AL517) was purchased from Research and Diagnostic Systems, Inc. (Minneapolis, USA). BD Matrigel Matrix (#354234) was procured from BD Biosciences, (San Jose, CA). Fibronectin (#33016015), Pierce™ BCA Protein Assay Kit, (#23225), Invitrogen fetal bovine serum exosome-depleted, One Shot™ format (#A2720803), Precision Plus Protein Dual Color Standards (#161-0374) from Bio-Rad (California, USA), High-Capacity cDNA Reverse Transcription Kit (Applied Biosystems™; #4368814), TRIzol RNA Isolation Reagent (#AM9738) were procured from Thermo Fischer Scientific, (Waltham, USA). ExoEnrich™ (#PEC-50), ExoLyseP™ (#PEL-25P) were purchased from ExoCan Healthcare Technologies Pvt. Ltd. (Pune, India). ECL-substrate (#SC-2048) from Santa Cruz Biotechnology Inc. (California, USA). All the antibodies were procured from Santacruz Biotechnology Inc. and Sigma (St. Louis, USA) (Additional file 1: Table S1) and oligos used in the study were procured from Eurofins. Scientific (Additional file 1: Table S2). Millipore PVDF membrane (#HVLP04700), RNAse A (#P4170), propidium iodide (PI; #R6513), PKH67 Green Fluorescent Cell Linker Kit for general cell membrane labeling (#PKH-67GL), DAPI (4',6-diamidino-2-phenylindole; #D-9542) and all other reagents unless specified were procured from Sigma.

### Cell culture

Cervical cancer cell lines were maintained in DMEM/MEM supplemented with 10% FBS as contamination-free cultures, supplemented with 1X antibiotic and antimycotic solution. HUVEC were maintained in endothelial basal medium supplemented with a cocktail of growth factors (1% glutamine and ECGS) provided by the manufacturer. Cells from passages 3 to 7 were used and grown on fibronectin-coated plates, in a humidified incubator at 37 °C with 5% CO_2_.

### Isolation of exosomes from cervical cell culture conditioned medium

Exosomes were isolated using commercially-available kit, ExoEnrich™ as described previously [24]. Briefly, filtered cell culture conditioned medium (4 ml) derived from cervical cancer cells (9 × 10^5^) cultured in 100 mm plate containing 10% exosome-depleted FBS for 4 days were used for exosome isolation. Conditioned medium was centrifuged at 2000 rpm for 10 min to remove dead cells followed by centrifugation at 5000 rpm for 30 min to pellet down remaining cellular debris. Exosome pellet was washed with phosphate buffered saline (1X PBS). Exosome preparations were quantified using BCA protein estimation reagent as per manufacturer’s protocol and used for downstream analysis or stored at − 80 °C until further use.

### Transmission electron microscopy (TEM) of cervical cancer exosomes

TEM analysis of exosome samples was performed according to previously published reports with minor modifications [38, 39]. Freshly isolated cervical cancer exosomes were resuspended in 30 µl of 1X PBS containing 2% paraformaldehyde. Exosomes were prepared for TEM inspection by adsorbing onto Formvar carbon-coated nickel grid for a time period of 1 h. The grids were fixed by 2.5% glutaraldehyde in 0.1 M sodium cacodylate, pH 7.6 for 10 min. After rinsing with sterile distilled water, the grids were contrasted using uranyl-oxalate solution at pH-7 for 5 min, air-dried for 5 min and examined with a JEOL 2100F transmission electron microscope (JEOL Ltd., Tokyo, Japan) operated at 100 kV.

### Transcript analysis by reverse transcriptase (RT)-PCR

Target cells or exosomes were harvested as per experimental protocols and RNA was isolated using TRIzol RNA Isolation Reagent as described earlier [24, 36]. Isolated RNA (2–10 µg/10 µl) was treated with DNase I (1U) for 30 min at 37 °C followed by DNase I inactivation using 2.5 µl of 25 mM EDTA solution and incubation at 65 °C for 15 min. Quantification was performed using NanoQuant Plate™ (Tecan). A minimum of 2 µg of sample RNA was used for cDNA synthesis in a 20 µl reaction using High-Capacity cDNA Synthesis Kit. PCR was performed for amplification of respective genes CD31, VEGF-A, VEGF-B, angiopoietin-1, angiopoietin-2, and osteopontin on Veriti Thermal Cycler Pro from Applied Biosystems in a 10 µl reaction system. The PCR reaction proceeded as follows: 95 °C for 2 min, 35 cycles including denaturation at 95 °C for 30 s, annealing that varied in range of 56–59 °C for 40 s, polymerization at 72 °C for 10 min followed by final extension of 10 min at 72 °C. Primer sequence with annealing temperature is described in Additional file 1: Table S2. All quantifications were normalized to the level of GAPDH transcripts which was used as input control.

### Immunocytochemistry (ICC) for endothelial marker CD31

Cellular localization of CD31 was determined by ICC as described earlier [40] with minor modifications. HUVEC were seeded on coverslips in 6-well plates at a density of 5000 cells/well. Next day, medium was removed and cells were fixed in 4% paraformaldehyde for 20 min and permeabilised with 0.2% Triton X-100 in 1X PBS. Cells were blocked with 5% BSA in 1X PBS for 1 h. Cells were incubated with primary antibodies (Additional file 1: Table S1) for 3 h followed by incubation with fluorescent-tagged secondary antibodies for 1 h. Counter-staining was done with DAPI (50 ng/ml). Finally, the coverslips were mounted on a microscope slide with Fluor mount as mounting medium. Preparations were visualized using a ZEISS Axio Imager Z2 microscope (Oberkochen, Germany).

### Tube formation assay for measuring angiogenic response

Tube formation assay was performed to assess the angiogenic potential of HUVEC cells in a 96-well plate as described previously [41] with minor modifications. Wells were coated with Matrigel (70 μl/well) and allowed to solidify for 1 h. HUVEC (10,000 cells/well) were plated into each well in HUVEC basal medium (100 μl/well) containing 2% FBS (reduced serum) in the absence or presence of 50 μg/ml of exosomes prepared from different cervical cancer cell lines and incubated at 5% CO_2_ at 37 °C. After 6 h, wells were imaged for tube formation representing HUVEC angiogenesis on a phase-contrast microscope (100X magnification) on Eclipse Ti2 (Nikon, Tokyo, Japan) and angiogenesis-related parameters were quantified from three random non-overlapping fields. Tube structures were analyzed by ImageJ software (Angiogenesis Analyser plugin version 1.51 s; National Institutes of Health, Bethesda, MD, USA). HUVEC seeded on Matrigel reorganized into capillary-like structures, which connected at certain points referred as nodes and formed mesh-like polygonal structures. These nodes represented the focal points for the branches to arise. A branching point was defined as a node connected to 3 different line segments and indicated either a new vessel sprout (branching) or two separate vessels fusing into one (anastomosis) [41]. The number of branch nodes and branches was estimated as a measure of angiogenic response.

### Wound healing assay for estimation of endothelial cell migration

To assess the endothelial cell migration in vitro wound healing assay was performed as described previously [42] with minor modifications. HUVEC (3 × 10^4^ cells/well) were grown to confluence in a 24 well plate. Before inducing a scratch, the complete medium was replaced with basal medium supplemented with 2% serum to reduce cell proliferation and cells were cultured for a period of 12–15 h. Next day, a “'scratch wound” was introduced in the confluent monolayer by scratching with a 200 μl pipette tip. Cells were washed with 1X PBS twice to remove cell debris and detached cells from the wound. Cells were incubated in fresh basal medium with reduced serum in absence or presence of cervical cancer exosomes. The wound was photographed for 0 h and after 24 h, cells were imaged under a phase contrast microscope and checked for presence of migrated cells in the wound area. Cells migrated into the wound area were calculated and plotted using ImageJ.

### Exosome uptake assay

Exosomal uptake studies were performed as described earlier [43] with some minor modifications. HUVEC (2 × 10^4^) were seeded onto fibronectin pre-coated (5 μg/ml) glass cover slips placed inside a 24-well plate. Cells were allowed to grow for 24 h. Cells were incubated with cervical cancer exosomes labelled with PKH-67 as per manufacturer’s protocol. For exosome labelling, exosomes (~ 100 μg) in pellet were resuspended in 1 ml of diluent C and mixed with 2 μl of PKH-67 in 1 ml of diluent C followed by an incubation of 5 min. Subsequently, the labelling reaction was stopped by adding an equal volume of Dulbecco’s modified eagle medium supplemented with 2% exosome depleted FBS. Labelled exosomes were again re-isolated using ExoEnrich™ kit as per manufacturer’s instructions at 3000 rpm for 20 min. The labelled exosome pellets were washed twice with 1X PBS to remove unbound dye and resuspended in a total of 50 μl of 1X PBS. PKH-67 labelled exosomes were quantified again using BCA protein estimation kit and a total of 50 μg of labelled exosomes were incubated onto PBS washed HUVEC monolayer in 0.5 ml of complete endothelial basal medium for 6 h. Treated HUVEC cells were washed with 1X PBS thrice and fixed using 4% paraformaldehyde for 10 min followed by rehydration using 1X PBS for 5 min. Nuclei were counter-stained with DAPI (50 ng/ml). Images were acquired using a Leica SP8 Spectral Confocal Laser Scanning Microscope at 630X magnification (Wetzlar, Germany).

### Isolation of cellular and exosomal proteins and immunoblotting

Total cellular proteins were isolated from cervical cancer cells as described earlier [44]. Briefly, 1 × 10^6^ cells were re-suspended in the cell lysis buffer [20 mM Tris (pH 7.4), 250 mM NaCl, 2 mM EDTA (pH 8.0), 0.1% Triton X-100, 0.01 mg/ml aprotinin, 0.005 mg/ml leupeptin, 0.4 mM PMSF, and 4 mM Na_3_VO_4_]. Lysates were spun at 14,000 rpm in a microfuge for 10 min. to remove insoluble material and clear supernatant for each sample was collected. Total exosome proteins were isolated using ExoLyseP™ as described earlier [24]. The concentration of total proteins was determined by BCA spectrophotometric method. Proteins were stored in small aliquots at − 80 °C till further use. Proteins (50 μg/lane) were resolved in 10% polyacrylamide gel using 2X Laemmli buffer (100 mM Tris–HCL pH 8.0, 20 mM EDTA pH 8.0, 4% SDS, 20% glycerol, 10% β-mercaptoethanol, 0.02% bromophenol blue) and transferred to PVDF membranes (0.45 µm; Millipore) by wet transfer method at 25 V for 2 h or using a G2 Fast Blotter (Thermo Scientific) in semi-dry conditions for 10 min. Membranes were blocked with 5% (w/v) bovine serum albumin in tris-buffered saline supplemented with Tween 20 (0.1%) (.TBST) for 2 h, and incubated with pre-standardized dilution of primary antibodies in TBST overnight at 4 °C. Antibodies and their specific dilution in the blocking solution used in the study are described in Additional file 1: Table S1. Membranes were washed with tris buffer saline (TBS) and were incubated with horseradish peroxidase (HRP-conjugated) secondary antibodies diluted in 5% BSA in TBS-Tween (0.1%) for 60 min. at room temperature. The blot was subsequently re-probed with β-actin and the absence of leftover signal following stripping was ascertained before the reprobing cycle. The western blot membranes were stripped at each interval using mild stripping buffer (1.5% glycine, 0.1% SDS, 1% Tween-20 pH-2.2) for 15 min. at room temperature followed by re-blocking. Immuno-active bands were detected on a (Bio-Rad ChemiDoc-XRS, IL, USA) imaging system or under Amersham Imager 600 (GE Life Sciences ABI, Sweden) after 5 min treatment of the blot with enhanced chemiluminescent substrate Luminol detection kit. β-actin expression was used as an internal control. The quantitative densitometric analysis of the bands was performed using ImageJ software.

### Statistical analysis

The data analysis was performed using the Microsoft Excel. Statistical significance of difference between the 2 test groups was analyzed by the Student’s *t*-test. In all cases, *p* value ≤ 0.05 was considered as significant.

## Results

**Fig. 1:**
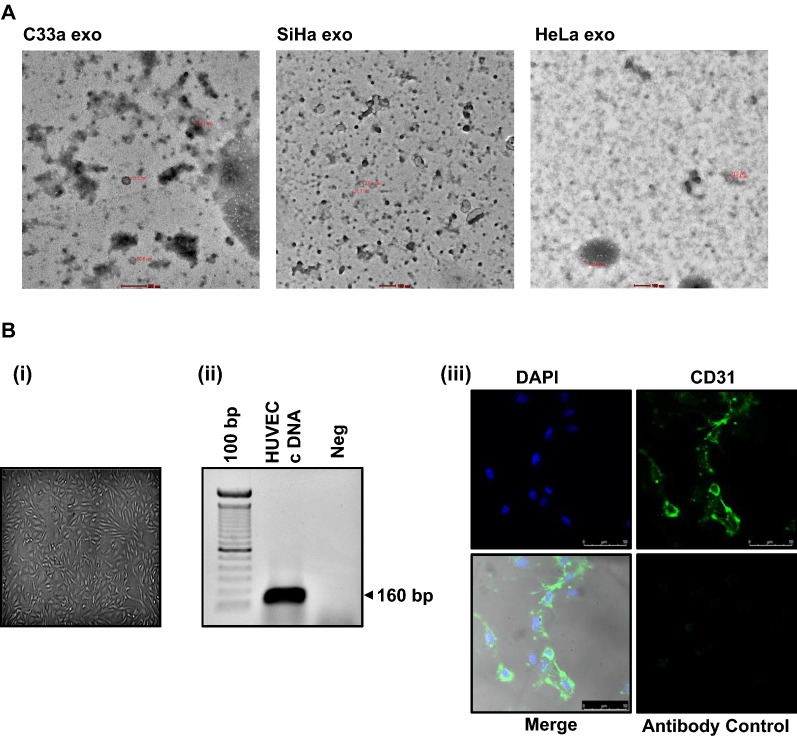
Characterization of cervical cancer exosomes and HUVEC. **A** Morphometric and particle size measurement of cervical cancer exosomes. Representative transmission electron photomicrographs of cervical cancer exosomes. Marking on the images indicate respective diameter size of visualized exosomes. **B** Characterization of HUVEC. **(i)** Photomicrograph of HUVEC culture showing typical ‘cobblestone’ morphology (magnification—100×) **(ii)**. Expression of CD31 transcripts in HUVEC. RT-PCR-based analysis of CD31 endothelial cell marker. **(iii).** Cytoplasmic localization of CD31. Representative immunofluorescence photomicrograph of HUVEC stained with Alexa Flour-488 labelled anti-CD31 (green), and counter-stained with DAPI (blue) (magnification—200×)

To decipher the impact of cervical cancer exosomes on endothelial cell functions, we first characterized the exosomes for their size and morphology; and HUVEC for their culture characteristics and expression of identification marker, CD31. Two HPV- positive (SiHa and HeLa) and one HPV-negative (C33a) cell lines were used for isolation of the exosomes. Exosomes isolated showed cup-shaped morphology of vesicles as observed under an electron microscope (Fig. 1A). Identified structures were bilayer vesicles, homogenously distributed in the diameter size range of 22–133 nm. Identity of endothelial cells was reconfirmed by ‘cobblestone morphology’ in culture (Fig. 1B(i)) and by examining the presence of endothelial cell-specific marker (CD31) in a RT-PCR reaction. PCR revealed an expected amplicon size of 160 bp corresponding to amplification of CD31 cDNA (Fig. 1B(ii)). Fluorescence microscopy performed on endothelial cells revealed homogenous and a specific distribution of CD31 in HUVEC cytoplasm (Fig. 1B(iii)).

### Cervical cancer exosomes stimulate tube formation in endothelial cells

**Fig. 2:**
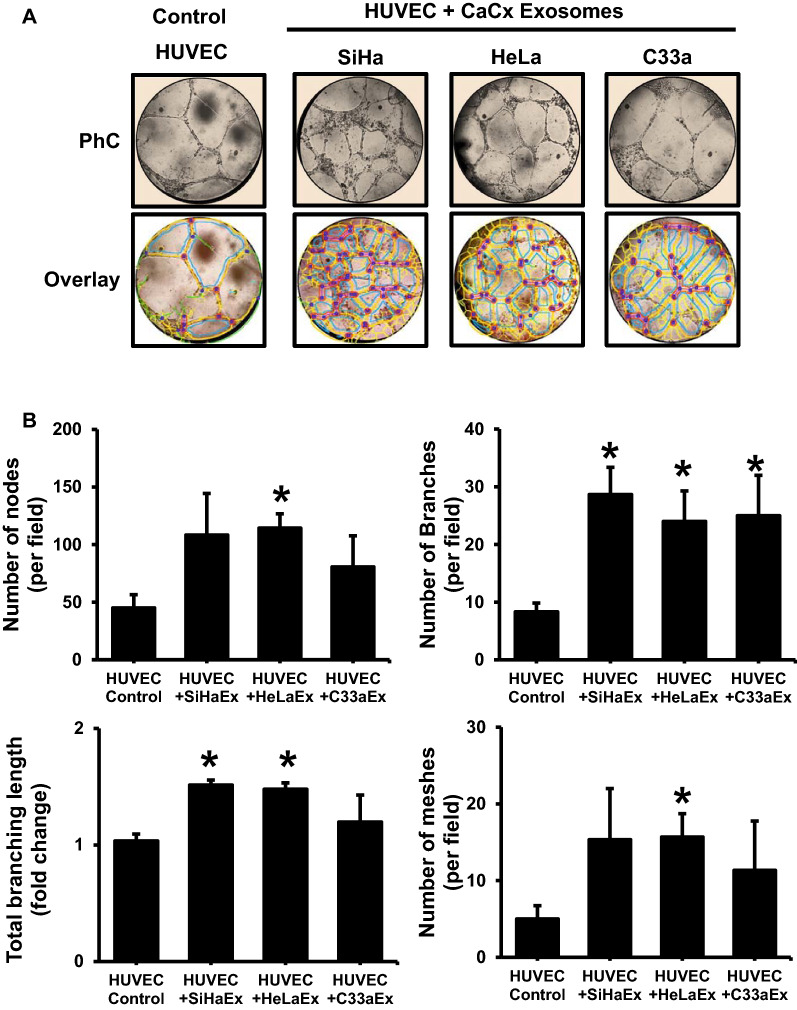
Effect of cervical cancer exosomes on endothelial cell vascularization. **A** Representative phase-contrast photomicrographs (magnification—100×) of endothelial tubular networks in Matrigel after 6 h in HUVEC co-incubated with exosomes (50 μg/ml) of different cervical cancer cell lines (upper row). Lower panels show corresponding quantitative evaluation of images by ImageJ Angiogenesis Analyzer plugin for meshes, nodes and tubes. **B** Effect of cervical cancer exosomes on total angiogenic profile. Cumulative data on number of nodes, branches, branch length and meshes of a representative experiment out of three independent experiments. Values are represented as mean ± s.d. (indicated as error bar). *p value ≤ 0.05 with respect to untreated HUVEC control

To understand the influence of exosomal cargo on endothelial cell functions, HUVEC were co-cultured for 6 h in the presence of exsosomes (50 μg/ml) derived from HPV-positive (SiHa and HeLa) and HPV-negative (C33a) cervical cancer cells in a matrigel tube formation assay. Microscopic examination of the cultures showed development of a denser endothelial tubular network compared to the control (Fig. 2A). Quantitative analysis revealed a higher number of nodes, tube-like structures and meshes (branches) in HUVEC treated with exosomes (Fig. 2B). The increase in number of nodes was nearly twofold, whereas number of branches increased approximately by threefold. As compared to HPV-negative C33a exosomes, relatively higher values were recorded for different angiogenic parameters like nodes, total branch length and number of meshes for HPV-positive cervical cancer exosomes (SiHa, HeLa); however, the trend did not cross the limits of statistical significance.

### Cervical cancer exosomes promote endothelial cell migration

**Fig. 3:**
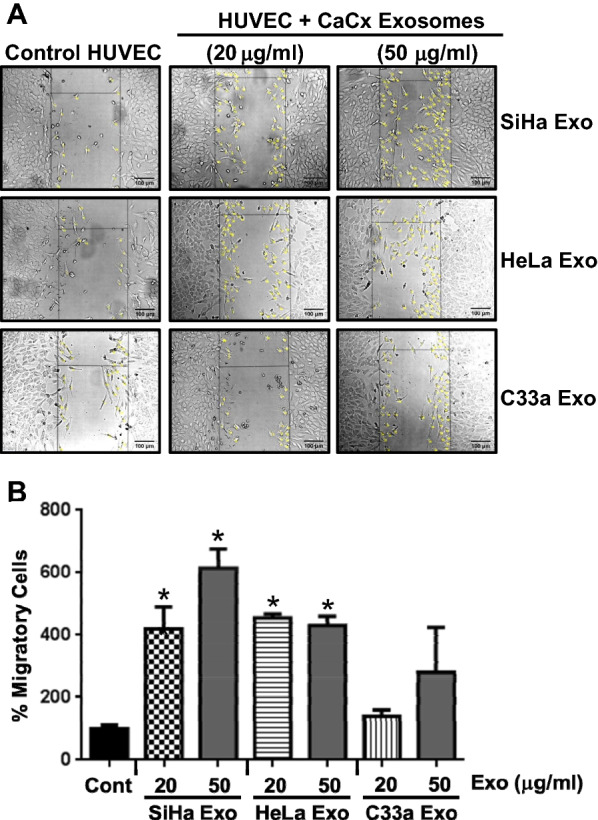
Effect of cervical cancer exosomes on endothelial cell migration. **A** Representative phase-contrast photomicrographs of HUVEC cells grown in presence of the exosomes after 24 h (magnification—100×). **B** Cumulative data of percent migratory cells of a representative experiment. Values are represented as mean ± s.d. (indicated as error bar). *p value ≤ 0.05 with respect to untreated HUVEC control cultures

Next, we performed the wound healing assay to check the migratory property of HUVEC under the influence of cervical cancer exosomes. The assay showed increase in number of migrated HUVEC treated with the exosomes into wound area as compared to the untreated control HUVEC (Fig. 3A). Quantitative analysis of migrated cells revealed higher endothelial cell migration in HUVEC treated with SiHa and HeLa cell-derived exosomes at 20 μg/ml (*p* value: 0.022 and 0.0012) and at 50 μg/ml concentrations (*p* value: 0.007 and 0.0043) (Fig. 3B). HUVEC treated with C33a exosomes also showed an upward trend in number of migrated cells; however, the increase did not achieve required statistical strength even at 50 μg/ml concentration. Similarly, the marginal increase in endothelial migration induced by exosomes from HPV-positive cells over HPV-negative was noted but it lacked statistical significance. In addition, a marginal dose-dependent increase in the proportion of S-phase cells was noted in response to different cervical cancer exosome treatment endothelial cells (Additional file 2: Figure S1).

### Cervical cancer exosomes are internalised in endothelial cells

**Fig. 4:**
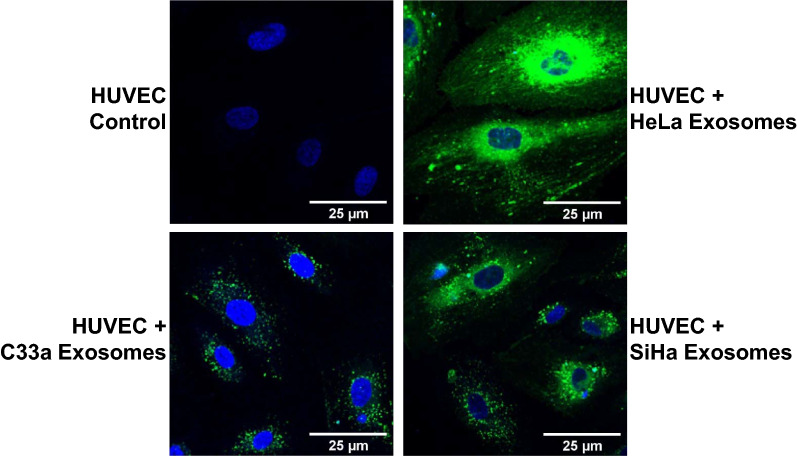
Assessment of exosomal uptake in endothelial cells. Fluorescence photomicrographs showing HUVEC after 6 h incubation with SiHa, HeLa, and C33a-derived exosomes (50 μg/0.5 ml) labelled with PKH-67 dye (green). HUVEC were fixed in cold methanol and nuclei were counterstained with DAPI (blue; magnification—630×)

To understand the physical interaction and uptake of exosomal cargo from cervical cancer cells by endothelial cells, HUVEC were incubated with PKH-67 labelled cervical cancer exosomes for 6 h. Fluorescence microscopy showed internalization of PKH-67 labelled exosomes in HUVEC irrespective of the cell of their origin (Fig. 4). A comparative analysis among different types of exosomes showed a differential exosome uptake. HUVEC showed highest uptake efficiency of HeLa exosomes, followed by SiHa exosomes. The uptake efficiency was the lowest for C33a exosomes.

### Cervical cancer exosomes increase PTCH1 protein level in endothelial cells

**Fig. 5:**
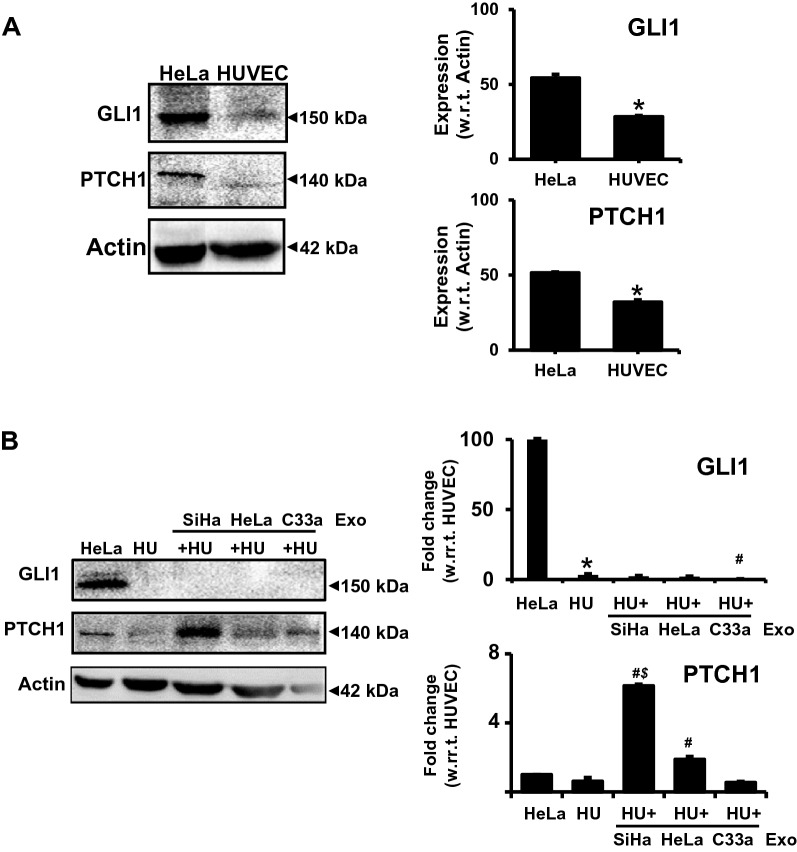
Effect of cervical cancer exosomes on levels of GLI1 and PTCH1 in endothelial cells. Immunoblots showing endogenous levels (**A**) of GLI1 and PTCH1 in total cellular proteins (50 μg/lane) from untreated and exosome-treated HUVEC (50 μg/ml) (**B**) in comparison to HeLa cells (positive control). Right panels show change in protein levels of PTCH1 and GLI1 in HUVEC in response to cervical cancer exosomes. Densitometric values of the bands in comparison to untreated HUVEC are represented as mean ± s.d. (indicated as error bar). *p value ≤ 0.05 with respect to total proteins from HeLa cells, ^#^*p* value ≤ 0.05 with respect to total proteins from untreated HUVEC control, ^$^*p* value ≤ 0.05 with respect to total proteins from HeLa exosome-treated HUVEC

Cervical cancer exosomes were found to carry several upstream components of Hh-GLI signaling [24], out of these PTCH1 showed the highest protein content. Therefore, next we examined the levels of PTCH1 and GLI1 in exosome-treated endothelial cells. Our immunoblotting data revealed that HUVEC showed minimal endogenous level of PTCH1, whereas GLI1 level was found inconsistent (Fig. 5A). Treatment with exosomes derived from HPV-positive cells resulted in increased PTCH1 level in the HUVEC (Fig. 5B). Among these, SiHa exosome-treated HUVEC showed the highest level of PTCH1. On the other hand, no notable change was observed in the expression levels of GLI1 in these treated HUVEC.

### Cervical cancer exosomes increased transcript levels of angiogenesis-related downstream genes of Hh-GLI signaling

**Fig. 6:**
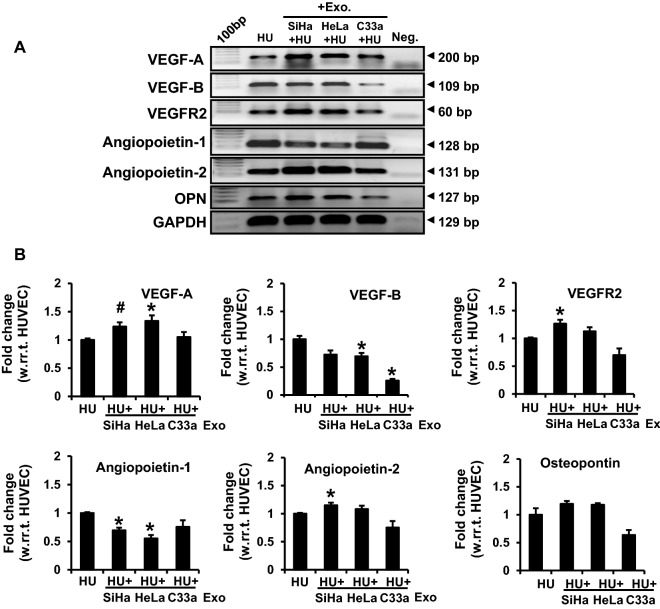
Transcript analysis of selected angiogenesis-related downstream genes of Hh-GLI signaling pathway in exosome-treated endothelial cells. **A** Gel images showing amplicons of different RT-PCR performed on cDNA prepared from 2 μg of total RNA isolated from untreated HUVEC or treated with the exosomes (50 μg/ml). **B** Densitometric values of bands normalised to GAPDH are represented as mean ± s.d. (indicated as error bar). *p value ≤ 0.05 with respect to the transcript level in untreated HUVEC control. ^#^p value 0.059. HU: HUVEC

Further, the transcript levels of angiogenesis-related downstream genes VEGF-A, VEGF-B, VEGFR2, angiopeotin-1, angiopeotin-2, and osteopontin were measured in the exosome-treated HUVEC. HUVEC treated with HPV-positive cervical cancer exosomes showed an increased transcript level of VEGF-A, VEGFR2, and angiopoietin-2 (Fig. 6). Notably, these HUVEC showed a concomitant decrease of VEGF-B, and angiopoietin-1 transcripts. On the other hand, HUVEC treated with C33a exosomes also showed reduced VEGF-B, but there was no corresponding increase in VEGF-A or angiopoietin-2. VEGFR2 and osteopontin showed a downward trend in the treated HUVEC, though statistically the difference was not significant. Osteopontin level, however, did not change significantly in HUVEC treated with HPV-positive exosomes.

### Cervical cancer exosomes lacked VEGF transcripts and proteins

**Fig. 7:**
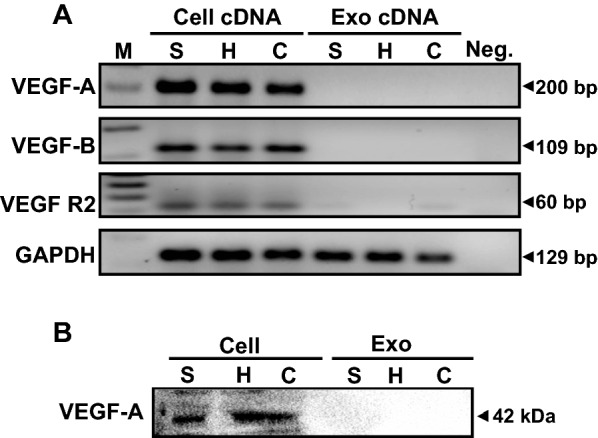
Analysis of VEGF transcripts and proteins in cervical cancer cells and their respective exosomes. **A** Gel images showing amplicons of the RT-PCR performed on cDNA prepared from 2 μg of total RNA isolated from cervical cancer cell lines and their exosomes. **B** Immunoblots of total cellular and exosomal proteins (50 μg/lane) probed for VEGF-A. M: marker, S: SiHa, H: HeLa, C: C33a

To rule out direct supplementation of VEGF transcripts/proteins from cervical cancer exosomes, the levels of VEGF transcripts and proteins were examined in total RNA and proteins isolated from exosomes and compared with the corresponding parental cells. RT-PCR results showed prominent presence of VEGF-A, VEGF-B and VEGFR2 transcripts in parental cervical cancer cells irrespective of their HPV status (Fig. 7A). However, none of the exosome preparations, showed presence of either VEGF-A, VEGF-B or VEGFR2 transcripts. Similarly, immunoblotting experiments showed presence of VEGF-A in all three cervical cancer cell lines (Fig. 7B). Yet, VEGF-A protein was not detectable in any of the exosomes used in the present study.

## Discussion

We aimed this investigation to decipher the physiological relevance of tumor-derived exosomes in the angiogenic response with respect to HPV infection status of cervical cancer cells. Herein, we showed cervical cancer exosomes possessed pro-angiogenic factors that readily stimulated tube formation and enhanced branching. Exosome-treated HUVEC showed higher frequency of node formation. These exosomes increased the endothelial cell migration. Exosomes from HPV-positive cells, however, showed higher angiogenic potential and higher rate of cellular uptake by endothelial cells compared to HPV-negative exosomes. Investigation of underlying mechanisms responsible for the angiogenic potential of HPV-positive exosomes revealed increased PTCH1 and VEGF-A, along with VEGFR2 and angiopoietin-2 expression. However, the exosomes did not supplement VEGF-A transcripts or proteins to its elevated levels in endothelial cells. Exosomes used here were homogenous in size and showed morphology consistent with their known appearance. These exosomes expressed characteristic exosome-specific markers as described in our previous report [24].

Our observation that cervical cancer exosomes could induce pro-angiogenic response in HUVEC was witnessed and reported recently in cervical [21, 22, 45] and other cancers [46–48]. Thus, suggesting that pro-angiogenic effect seen by us and others is not a cell culture artifact. Recently, tumor-derived exosomes were shown to induce cytotoxicity against HUVECs [49]. However, induction of apoptosis in the endothelial cells by tumor-derived exosomes is not a universal phenomenon. Tumor exosomes are primarily known for their non-cytotoxic, pro-tumorigenic and pro-angiogenic response in cervical cancer [21, 22], glioblastoma [48], head and neck cancer [47], and ovarian cancer [46]. In our case, we observed no notable cytotoxicity of cervical cancer exosomes on endothelial cells in present or in our earlier study [24]. There was no remarkable change in the cell morphology and proliferation rates of endothelial cells in culture (Additional file 2: Figure S1), which was depictive of non-cytotoxic nature of these exosomes.

Using a panel of cell lines with different HPV status, we showed for the first time that exosomes derived from HPV-positive cervical cancer cells possessed stronger vascularity promoting nature. High vascularity co-relates with poor clinical outcome of cervical cancer patients [50]. The mechanism by which tumor-derived exosomes execute pro-angiogenic effect is poorly defined. Recent reports suggest specific role of exosomal microRNA cargo. MicroRNA miR-221-3p present in cervical cancer exosomes promoted angiogenic response [21, 45]. Further, HPV oncogenes were shown to influence exosomal microRNA cargo by reducing level of anti-angiogenic miR-377 in microvesicles thereby promoting endothelial cell proliferation, migration, and tube formation [51]. In contrast, a recent study carried on HeLa exosomes showed breakdown of vascular integrity and endothelial cell permeability, essential for neo-angiogenic response, by triggering endoplasmic reticulum stress leading to metastasis. The process was found microRNA-independent [52]. Therefore, mechanisms other than microRNA cargo may contribute substantially to the angiogenic response of cervical cancer exosomes.

Endothelial cell migration is essential for angiogenesis [53]. We observed that cervical cancer exosomes promoted endothelial cell migration. Moreover, HPV-positive exosomes particularly of SiHa cells induced more migration as compared to HPV-negative C33a cells. The underlying reasons for this enhanced migratory potential of HPV-positive exosomes are not known. Expression of HPV oncogenes in primary keratinocytes upregulated the secretion of pro-angiogenic factors, interleukin-8 and VEGF [54]. Conditioned medium from HPV16 E6E7 expressing keratinocytes was shown to stimulate proliferation and migration of human microvascular endothelial cells [14]. Cervical cancer exosomes were shown to promote endothelial migration and the effect was mediated through miR-221-3p [20, 45]. Similar endothelial migration was seen with exosomes derived from different epithelial cancer cells like head and neck cancers [47, 55, 56], melanoma [57] and ovarian cancer [58]. In view of these observations, we speculate that HPV-positive exosomes are likely to carry a higher content of pro-angiogenic mediators.

Endothelial cell proliferation is an integral component of pro-angiogenic response [59]. HeLa cells are known to release serine protease tissue Kallikrein that induced endothelial cell proliferation [60]. Incidentally, multiplex proximity extension assays on exosomal proteome showed presence of Kallikrein in human milk and prostate cancer exosomes [61], which suggests that pro-angiogenic proteins like Kallikrein may be directly exported through exosomes. Recently, CaSki and HeLa exosomes enhanced DNA synthesis activity and colony formation in endothelial cells [22]. However, lncRNA-TUG1 was shown to mediate the effect. Enhanced endothelial cell proliferation is also reported in colorectal cancer via export of M-phase-related mRNA in microvesicles [62]. Increased endothelial cell population in S and G2/M phase was observed in response to hypoxic exosomes of esophageal squamous cell carcinoma [63]. Proliferation of endothelial cells was reported in response to ovarian cancer exosomes using direct cell counting [46]. Therefore, exosomal cargo can directly promote endothelial cell proliferation. However, in our experiments, cervical cancer exosomes only marginally influenced endothelial cell population cycling in S/G2/Mphase (Additional file 2: Figure S1).

Our confocal data showed that cervical cancer exosomes readily get internalized in the endothelial cells, though the efficiency of uptake differed among exosomes from HPV-positive and HPV-negative cells. Higher degree of internalization was noted for HeLa exosomes in comparison to uptake of SiHa and C33a exosomes. Previously, uptake of HeLa exosomes was reported at 4 h [64], whereas, uptake of SiHa exosomes was described at 48 h [45]. These analyses, however, lacked comparative value due to different experimental conditions. Long incubations fail to register the early differences in uptake as endothelial cells show a tendency to internalize lipid-rich vesicles and accumulate them gradually [64, 65]. Therefore, shorter incubation periods like 6 h in our study were extremely informative to decipher the differences in uptake of exosomes of HPV-positive and HPV-negative cells. However, the underlying reasons of differential uptake among exosomes of different cervical cancer cells are not known. Regardless of the higher uptake, HeLa-derived exosomes showed similar or lesser pro-angiogenic effects than SiHa exosomes. These observations are suggestive of quantitative and/or qualitative differences in the exosomal cargo of different HPV-positive and HPV-negative cervical cancer cells, which needed further investigation.

Earlier, we showed over-loading of Hh-GLI signaling components like PTCH1, Shh, and Ihh on cervical cancer exosomes regardless of the cell type. The expression level of PTCH1 was found to be consistently higher [24]. Here we observed that these exosomes induced elevated PTCH1 protein level in the endothelial cells. Resting endothelial cells in isolation normally failed to respond to Hh ligands. These cells reportedly lacked functional Hh-GLI transcriptional response and required paracrine secretions from fibroblasts [66]. However, in natural conditions whether these mediators are transported via exosomes is not known. The horizontal transfer of PTCH1 or its transcriptional upregulation via active Hh-GLI signaling could be the potential contributors to elevated PTCH1 levels in exosome-treated endothelial cells. Export of Shh through tumor exosomes has been reported recently in esophageal cancer [67]. However, currently there is no additional evidence thatcan support complementation of Hh-GLI components in endothelial cells. A functional Hh-GLI signaling is essentially required for tube formation during vasculogenesis [68] and can operate through both canonical Hh-GLI-mediated transcription and non-canonical signaling driven through PTCH1 [69]. Activation of Hh-GLI signaling through Shh induces expression of angiogenesis-related proteins including all VEGF isoforms, angiopoietin-1, angiopoietin-2 [70], VEGFR2 [71] and osteopontin [72], apart from upregulating expression of all three GLI isoforms and *ptch1* gene [73].

HPVE6-mediated, constitutively active Hh-GLI signaling is characteristic of cervical cancer cells [36]. Disruption of the Hh-GLI signaling in the tumor cells reduced their ability to induce angiogenic response of endothelial cells [71], which also required active Hh-GLI signaling. Our data showing increased transcripts of VEGF-A, VEGFR2 and angiopoietin-2, and decreased transcripts of angiopoietin-1 and VEGF-B along with higher protein level of PTCH1 are suggestive of plausible transcriptional upregulation of Hh-GLI signaling in endothelial cells. However, how Hh-GLI signaling operating in the cancer cells modulate the Hh-GLI signaling in the endothelial cells is not known. The expression levels of other downstream Hh-GLI targets like PTCH2 and SFRP1 will help in better understanding. In contrast, lack of GLI proteins in exosome-treated endothelial cells was intriguing. Induction of pro-angiogenic response and expression of downstream Hh-GLI target genes in the absence of detectable GLI1 protein, is not uncommon [74].

Mechanistically, VEGF family proteins represented by VEGF-A and VEGF-B prime endothelial cell proliferation and migration through interaction with VEGFR2 present on endothelial cells [75]. Though contribution of VEGF-B protein cannot be ruled out, VEGF-A is the primary ligand which regulates the pro-angiogenic response in the endothelial cells. VEGF-A was found upregulated/increased at the transcript level in exosome-treated HUVEC (Fig. 6A). Hence, we focused our study on VEGF-A. Incidentally, our data showed absence of VEGF-A in the cervical cancer exosomes. In line with our observation, ovarian cancer exosomes promoted VEGF expression and secretion in endothelial cells [46]. Angiopoietin-1 and angiopoietin-2 also contribute to angiogenesis, but the nature of their contributions is distinct. Angiopoietin-1 plays a key role in maintaining the integrity of existing vessels. Angiopoietin-2 is mainly secreted by endothelial cells at sites of active vascular remodeling, and is involved in tumor initiation [76]. Interestingly, we observed a reciprocal change in angiopoietin-1 and angiopoietin-2 transcripts in endothelial cells treated with cervical cancer exosomes and shifting of the angiopoietin balance towards angiopoietin-2. Vascular stability versus neo-angiogenesis largely depends on balance between angiopoietin-1 and angiopoietin-2 [77]. Therefore, shifting of this balance towards angiopoietin-2 may have an important role in early initiation and activation of neo-angiogenesis during malignant transformation. This is further supported by concomitant upregulation of VEGFR2, which is a specific isoform essentially required in endothelial cells of blood vessels for neo-vascularization [78]. Although, our data showed alterations in key angiogenic mediators at transcript level, further validation at protein level is required. Therefore, we consider this as a preliminary observation and leave the field open for others to pursue it further.

Lastly, our data confirmed that exosomal transcript or protein cargo did not contribute to the elevated VEGF detected in endothelial cells treated with cervical cancer exosomes. Cervical cancer cells express high level of VEGF both at transcript and protein level as seen in present study and reported previously by others [12]. HPV E6 plays an instrumental role in transcriptional upregulation of VEGF in cervical cancer cells [13]. Despite overexpression in tumor cells, no VEGF-A transcripts or proteins could be detected in cervical cancer exosomes irrespective of their HPV status. The presence of VEGF transcripts and proteins in exosomal cargo is not universal [17]. However, presence of VEGF in exosomes of selected malignancies like glioblastoma [79], melanoma [80] and multiple myeloma [81] have been reported. In contrast, absence of VEGF from the pro-angiogenic exosomal cargo has been reported [82]. In view of these observations, it seems that cervical cancer exosomes despite lacking VEGF transcripts and proteins, are well-enriched with bio-macromolecules which can sufficiently induce the angiogenic response in endothelial cells, independent of VEGF secretions from cervical cancer cells.

## Conclusion and future directions

We, therefore, conclude that cervical cancer exosomes, particularly, from high-risk HPV-positive cells are functionally active nano vesicles that promote angiogenic response in neighboring endothelial cells in a dose-dependent manner. Following their cellular uptake, these exosomes induce key angiogenic modulators, VEGF-A, VEGFR2 and angiopoietin-2, controlled by Hh-GLI-signaling. The study provides a novel exosome-mediated mechanism potentially favoring cervical angiogenesis and thus identifies exosomes as potential pharmacological targets against locally advanced metastatic cervical lesions.

## Supplementary Information

**Additional file 1: Table S1.** List of antibodies used for immunoblotting (IB) experiments. **Table S2.** List of primers used in the study along with their sequence and annealing temperatures.

**Additional file 2: Figure S1.** Effect of cervical cancer exosomes on endothelial cell cycle.

## Data Availability

The data sets used and or/analyzed during the current study are available from the corresponding author on reasonable request.

## Abbreviations

GLI: Glioma-associated oncogene
Hh: Hedgehog
HR-HPV: High risk human papillomavirus
HUVEC: Human umbilical vein endothelial cells
PTCH1: Patched 1
TEM: Transmission electron microscopy
VEGF: Vascular endothelial growth factor
