# Supplementary material for: Exosomes from cervical cancer cells facilitate pro-angiogenic endothelial reconditioning through transfer of Hedgehog–GLI signaling components
Source: Cancer Cell Int. 2021 Jun 24;21:319. doi: 10.1186/s12935-021-02026-3 (PMC8223267; doi:10.1186/s12935-021-02026-3)
Supplement: Supplementary file 1 — Additional file 1: Table S1. List of antibodies used for immunoblotting (IB) experiments. Table S2. List of primers used in the study along with their sequence and annealing temperatures. [file 12935_2021_2026_MOESM1_ESM.docx]

**Table S1: List of antibodies used for immunoblotting (IB) experiments.**

| **S. No.** | **Antibodies**  **(Mol. Wt.)** | **Source and Cat. No.** | **Origin and type** | **Dilution in IB** |
| --- | --- | --- | --- | --- |
|  | GLI-1 (D-1)  (150 kDa) | Santa Cruz  (sc-271075) | Mu monoclonal IgG_1_ | 1:3000 |
|  | VEGF (147)  (42 kDa) | Santa Cruz  (sc-507) | Rabbit polyclonal IgG | 1:3000 |
|  | Patched  (140 kDa) | Santa Cruz  (sc-6149) | Goat polyclonal IgG | 1:3000 |
|  | β-actin  (42 kDa) | Sigma  (A1978) | Mu monoclonal IgG_1_ | 1:5000 |
|  | Anti-mouse-HRP | Santa Cruz  (sc-2031) | Goat anti-mouse IgG | 1:5000 |
|  | Anti- Rabbit-HRP | Santa Cruz  (sc-2030) | Goat anti-rabbit IgG | 1:5000 |
|  | Anti- Goat-HRP | Santa Cruz  (sc-2033) | Donkey anti-goat IgG | 1:5000 |

**Table S2: List of primers used in the study along with their sequence and annealing temperatures**

| **Primer** | **Sequence** | **Amplicon size** | **Annealing temp.** | **References** |
| --- | --- | --- | --- | --- |
| **VEGF-A** | Forward: 5' CTTGCCTTGCTGCTCTACC 3'  Reverse: 5' CACACAGGATGGCTTGAAG 3' | 200 bp | 56 | [[1](#_ENREF_1)] |
|  |  |  |  |  |
| **VEGF-B** | Forward: 5’ AGCACCAAGTCCGGATG 3’  Reverse: 5’ GTCTGGCTTCACAGCACTG 3’ | 109 bp | 56 | [[1](#_ENREF_1)] |
|  |  |  |  |  |
| **VEGFR2** | Forward: 5’ ATTCCTCCCCCGCATCA’3  Reverse: 5’ GCTCGTTGGCGCACTCTT 3 | 60 bp | 58 | [[2](#_ENREF_2)] |
|  |  |  |  |  |
| **ANG1** | Forward: 5’ GGACAGCAGGAAAACAGAGC 3'  Reverse: 5' CACAAGCATCAAACCACCAT 3' | 128 bp | 57 | [[3](#_ENREF_3)] |
|  |  |  |  |  |
| **ANG2** | Forward: 5’ ACTGTGTCCTCTTCCACCAC’3  Reverse: 5’ GGATGTTTAGGGTCTTGCTTT 3 | 131 bp | 59 | [[4](#_ENREF_4)] |
| **OPN** | Forward: 5’ CGAGGTGATAGTGTGGTTTATGG 3’  Reverse: 5’ GCACCATTCAACTCCTCGCTTTC**-** 3’ | 127 bp | 62 | [[5](#_ENREF_5)] |
|  |  |  |  |  |
| **CD31** | Forward: 5’ ATTGCAGTGGTTATCATCGGAGTG  Reverse: 5’ CTCGTTGTTGGAGTTCAGAAGTGG | 140 bp | 58 | [[6](#_ENREF_6)] |
|  |  |  |  |  |
| **GAPDH** | Forward: 5’ GTCTCCTCTGACTTCAACAGCG  Reverse: 5’ ACCACCCTGTTGCTGTAGCCAA | 129 bp | 62 | [[7](#_ENREF_7)] |
|  |  |  |  |  |

**References:**

1. Niki T, Iba S, Tokunou M, Yamada T, Matsuno Y, Hirohashi S: **Expression of vascular endothelial growth factors A, B, C, and D and their relationships to lymph node status in lung adenocarcinoma**. *Clinical cancer research : an official journal of the American Association for Cancer Research* 2000, **6**(6):2431-2439.

2. Domingues I, Rino J, Demmers JA, de Lanerolle P, Santos SC: **VEGFR2 translocates to the nucleus to regulate its own transcription**. *PloS one* 2011, **6**(9):e25668.

3. Li Y, Dewar A, Kim YS, Dey SK, Sun X: **Pregnancy success in mice requires appropriate cannabinoid receptor signaling for primary decidua formation**. *eLife* 2020, **9**.

4. Park SW, Yun JH, Kim JH, Kim KW, Cho CH, Kim JH: **Angiopoietin 2 induces pericyte apoptosis via alpha3beta1 integrin signaling in diabetic retinopathy**. *Diabetes* 2014, **63**(9):3057-3068.

5. Jabbari N, Nawaz M, Rezaie J: **Bystander effects of ionizing radiation: conditioned media from X-ray irradiated MCF-7 cells increases the angiogenic ability of endothelial cells**. *Cell communication and signaling : CCS* 2019, **17**(1):165.

6. Hamilton C, Callanan A: **Secreted Endothelial Cell Factors Immobilized on Collagen Scaffolds Enhance the Recipient Endothelial Cell Environment**. *BioResearch open access* 2016, **5**(1):61-71.

7. Nabokina SM, Inoue K, Subramanian VS, Valle JE, Yuasa H, Said HM: **Molecular identification and functional characterization of the human colonic thiamine pyrophosphate transporter**. *The Journal of biological chemistry* 2017, **292**(40):16526.
