# Supplementary material for: Exosomes from cervical cancer cells facilitate pro-angiogenic endothelial reconditioning through transfer of Hedgehog–GLI signaling components
Source: Cancer Cell Int. 2021 Jun 24;21:319. doi: 10.1186/s12935-021-02026-3 (PMC8223267; doi:10.1186/s12935-021-02026-3)
Supplement: Supplementary file 2 — Additional file 2: Figure S1. Effect of cervical cancer exosomes on endothelial cell cycle. [file 12935_2021_2026_MOESM2_ESM.pdf]

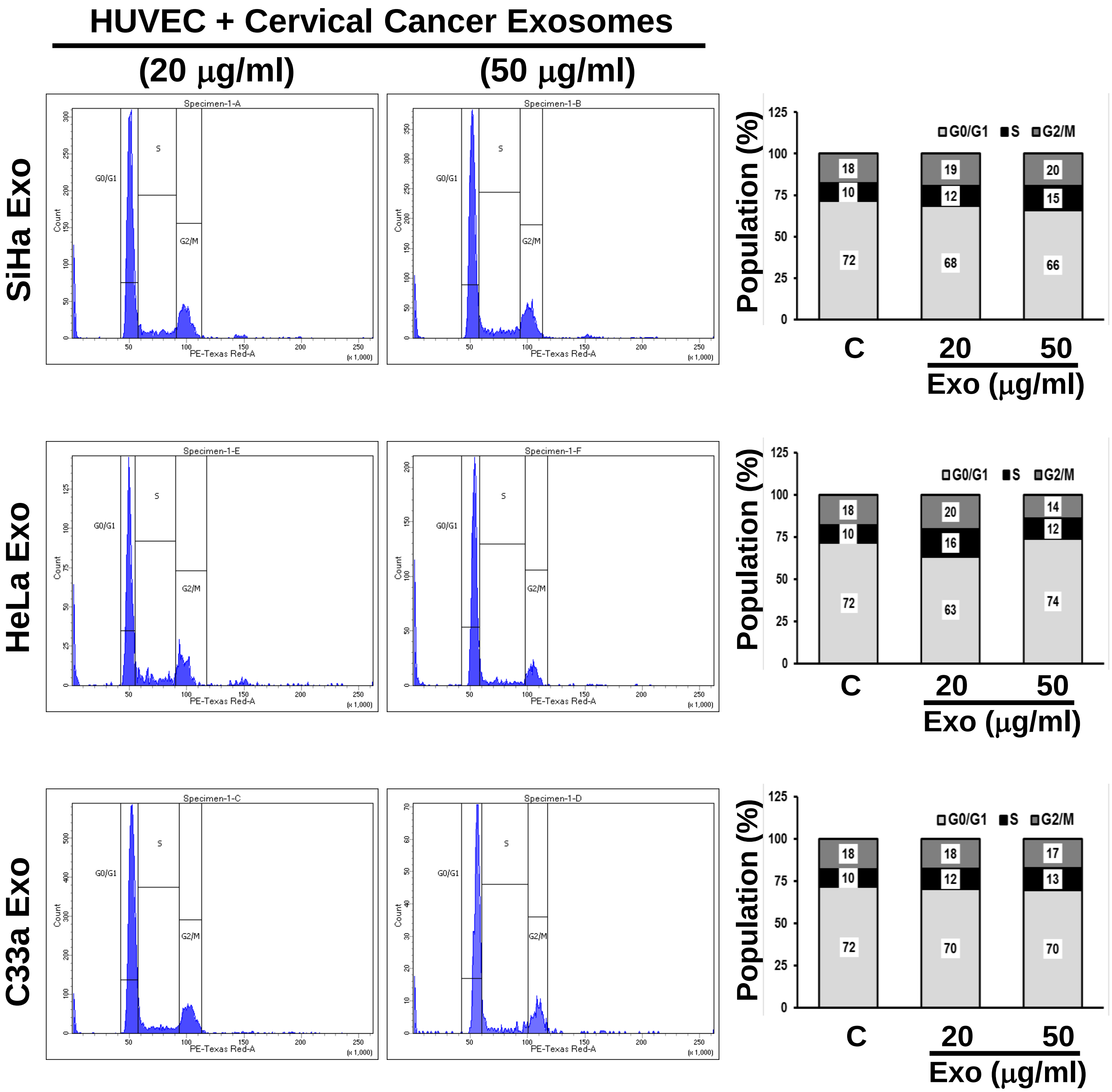

Control HUVEC

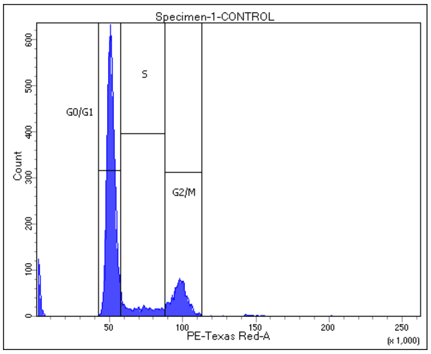

Fig. S1: Effect of cervical cancer exosomes on endothelial cell cycle. Histograms showing proportions of HUVEC incubated with indicated concentrations of cervical cancer exosomes in different phases of cell cycle. Right panels show phase-specific changes in the proportion of exosome-treated HUVEC. HUVEC cells (50,000 cells/well) were seeded in a 6-well plate in 10% exosome-depleted serum and cultured overnight. Next Day, cells were treated with cervical cancer exosomes for 24 hours. Subsequently, cells were harvested through trypsinization using 0.3 ml of 1X Tryipsin-EDTA solution (HiMedia) and fixed in 70% ethanol for 5 min. Fixed cells were rehydrated in 1X PBS for 10 min at RT and pelleted at 1200 rpm. Cell pellets were then resuspended in 1X PBS and treated with RNase A (10 μg/ml working concentration) for 5 min. Finally, PI (50 μl; 1mg/ml stock concentration) was added and cells were kept for 10 min in dark. Data was acquired on a BD FACS LSR Fortessa instrument (BD Biosciences; New Jersey, USA) using a 488 nm excitation laser. Cell cycle analysis was performed by BD DIVA software. A total of 10,000 events were acquired for each sample. For analysis, the cells were gated to exclude cell debris, cell doublets and cell clumps to identify the single cell population first using PI width vs. PI area. The gates were applied to the PI histogram plot.
